# Identification of Prognostic Biomarkers in Patients With Malignant Rhabdoid Tumor of the Kidney Based on mTORC1 Signaling Pathway-Related Genes

**DOI:** 10.3389/fmolb.2022.843234

**Published:** 2022-04-26

**Authors:** Chenghao Zhanghuang, Zhigang Yao, Haoyu Tang, Kun Zhang, Chengchuang Wu, Li Li, Yucheng Xie, Zhen Yang, Bing Yan

**Affiliations:** ^1^ Department of Urology, Kunming Children’s Hospital, Kunming, China; ^2^ Key Laboratory of Pediatric Major Diseases, Kunming Children’s Hospital, Kunming, China; ^3^ Department of Pathology, Kunming Children’s Hospital, Kunming, China; ^4^ Department of Oncology, Kunming Children’s Hospital, Kunming, China

**Keywords:** mTOR signaling pathway, malignant rhabdoid tumor of the kidney, therapeutic, prognosis, target drugs

## Abstract

**Background:** Malignant rhabdoid tumor of the kidney (MRTK) is an infrequent malignant tumor in childhood, accounting for approximately 2% of all childhood kidney tumors. Although the development of current treatments, the overall survival (OS) rate of MRTK patients is only 25%. The aim of this research was to explore the prognostic value of genes associated with the mTORC1 signaling pathway in MRTK.

**Methods:** The transcriptome data of MRTK samples were downloaded from the TARGET database. The 200 genes of HALLMARK_MTORC1_SIGNALING were downloaded from the Molecular Signatures Database (MSigDB). Furthermore, we applied gene set variation analysis (GSVA) to screen differentially expressed gene sets between the MRTK and normal samples. The 200 genes were combined with differentially expressed genes (DEGs) identified from differentially expressed gene sets. Then, a gene signature of mTORC1 pathway-related genes (mTRGs) was constructed in MRTK. The molecular mechanism of prognostic factors in MRTK was further analyzed using gene set enrichment analysis (GSEA). The target drugs based on these prognostic factors were explored from The Comparative Toxicogenomics Database (CTD). Moreover, six paired fresh tumor tissues and paraneoplastic tissues from children with MRTK were collected to validate the expressions of P4HA1, MLLT11, AURKA, and GOT1 in clinical samples via real-time fluorescence quantitative PCR and Western blot.

**Results:** A four-gene signature (P4HA1, MLLT11, AURKA, and GOT1) related to the mTORC1 pathway was developed in MRTK, which divided the MRTK patients into high-risk and low-risk groups. The patients with high-risk scores were strongly associated with reduced OS. Receiver operating characteristic (ROC) analysis indicated a good prediction performance of the four biomarker signatures. GSEA revealed that the mTOR signaling pathway was significantly enriched. The risk score was demonstrated to be an independent predictor for MRTK outcome. According to the correlation of tumor stem cell index and prognostic factors, the target drugs were obtained for the treatment of MRTK patients. Furthermore, the expressions of RT-qPCR and Western blot were consistent with RNA-sequencing data such that their expressions were significantly elevated in tumor tissues.

**Conclusion:** A total of four genes (P4HA1, MLLT11, AURKA, and GOT1) were screened as prognostic markers, further providing a new understanding for the treatment of patients with MRTK.

## Background

Malignant rhabdoid tumor of the kidney (MRTK) is a kind of malignant rhabdoid tumor (MRT), which often occurs in infanthood. It has high invasiveness, short survival rate, and fast metastasis, and up to 80% of patients have metastatic disease (Wang et al., 2020). It has been reported that the overall survival rate of patients with MRTK was only 25% (Yanagisawa et al., 2009). Therefore, MRTK has the worst prognosis of all renal tumors. Moreover, the prognosis was much worse for children younger than 6 months with distant brain metastasis (Tahir et al., 2019). Our previous studies have shown that the PI3K-AKT signaling pathway and microRNA-related proteins have a very high potential value for the diagnosis and treatment of MRTK. (Zhanghuang et al., 2021). However, the prognosis prediction of MRTK has not been fully clarified. Therefore, exploring new targets for MRTK is urgent.

mTOR is a serine/threonine protein kinase belonging to the phosphoinositide 3-kinase (PI3K)-related family, which interacts with several proteins to form two different complexes, called mTOR complex 1 (mTORC1) and mTOR complex 2 (mTORC2) (Zoncu et al., 2011). The core mTOR is mainly through insulin receptor (IR), insulin receptor substrate (IRS), type I phosphoinositide 3-kinase (PI3K), phosphoinositide-dependent protein kinase 1 (PDK1), and AKT-inducing kinase. The signaling cascade is mediated (Laplante and Sabatini, 2012). The loss of p53, a common event in cancer, promotes the activation of mTORC1 (Feng et al., 2005). The inhibition of mTORC1/mTORC2 blocked growth and induced catastrophic macropinocytosis in tumor cells (Kim et al., 2017; Srivastava et al., 2019). Significantly, there are no reports on the relationship between mTOR signaling pathway and MRTK.

In the present study, we successfully constructed a gene signature of mTORC1 pathway-related genes (mTRGs) and screened four genes (P4HA1, MLLT11, AURKA, and GOT1) as prognostic markers. The correlation between the tumor stem cell index and target drug was further explored, providing a new insight into further study of MRTK treatment.

## Methods

### Ethic Statement

All human studies performed in the current study were approved by the Medical Ethics Committee of Kunming Children’s Hospital before collection (approval number: 20190822002), and the guardians of the children signed informed consent forms.

### Data Collection

We retrieved the transcriptome data of MRTK from the TARGET database (https://ocg.cancer.gov/programs/target).

A total of 200 genes in the gene set of HALLMARK_MTORC1_SIGNALING were retrieved from the Molecular Signatures Database (MSigDB).

Clinical tissue specimen: The tissue specimens of six children with MRTK cancer and adjacent tissues in our hospital were selected. The relevant studies have been approved by the relevant ethics committee, and informed consent was signed by their families.

### Gene Set Variation Analysis

A total of 50 MRTK samples and six normal samples were applied to conduct GSVA, of which h.all.v7.2.symbols.gmt was considered to be the preset pathway. Then, we used the “limma” package to analyze the differences of GSVA scores between the MRTK and control groups. The screening conditions were |*t*-value| > 2 and *p*-value < 0.05. In the GSVA, the screened pathways with t-value > 0 were considered to be activated in the MRTK group, while screened pathways with *t*-value < 0 were considered to be activated in the normal group.

### Differentially Expressed Analysis of Activated Pathway in the Malignant Rhabdoid Tumor of the Kidney Group

The genes were extracted from the identified HALLMARK_MTORC1_SIGNALING pathway. Thereafter, we used the “limma” R package to perform the differential analysis for identifying the differentially expressed genes (DEGs) between the MRTK samples and normal samples. The screening conditions of DEGs were |log_2_FC| > 1 and *p*-value < 0.05. We combined the DEGs with 200 genes of the HALLMARK_MTORC1_SIGNALING gene set, obtaining the differentially expressed mTORC1 pathway-related genes (DE-mTRGs).

### Construction of an mTRG-Related Gene Signature

A total of 49 MRTK samples containing complete clinical information were applied to construct an mTRG-related gene signature. The univariate Cox regression analysis and Kaplan–Meier (K-M) survival curves were performed by employing the survival R package to identify the DE-mTRGs related to the overall survival (OS) of MRTK (*p* < 0.05). DE-mTRGs with *p* < 0.05 were input into a multivariate Cox regression analysis for the construction of an mTRG-related gene signature. The risk score of each MRTK sample is obtained as the following formula: Risk score = h0(t)*exp (*β*1*X*1+*β*2*X*2+…+*β*n*X*n). In this formula, *β* is the regression coefficient, and h0(t) is the benchmark risk function. A total of 49 MRTK samples were classified into high-risk and low-risk groups with the boundary of the median risk score. The K-M survival curve was generated via the “survdiff” functions of the R package to compare the OS of two risk groups. Thereafter, a time-independent receiver operating characteristic (ROC) curve was conducted to demonstrate the effectiveness of this mTRG-related gene signature for OS in MRTK patients.

The relationship between risk score and clinicopathological features was evaluated using the Chi-square test. Then, we executed univariate and multivariate Cox regression analyses to evaluate whether these clinicopathological features were independent predictors for RKT prognosis. A nomogram containing independent prognostic factors was generated using the “rms” R package, and the corresponding calibration plot was further established to evaluate the efficiency of the nomogram.

### Functional Enrichement Analysis

Gene set enrichment analysis (GSEA) of prognostic mTRGs was analyzed by the “clusterProfiler” R package (version 3.18.0) to explore the potential signaling pathways that were involved. The “limma” package was executed to screen the DEGs in high- and low-risk groups with the screening conditions being |log_2_FC| > 1 and *p*-value < 0.05. In addition, functional enrichment analyses including Gene Ontology (GO) and Kyoto Encyclopedia of Genes and Genomes (KEGG) analyses were further conducted using Metascape software based on the DEGs.

### Tumor Stem Cell Index Calculation and Drug Prediction for Malignant Rhabdoid Tumor of the Kidney

The tumor stem cell index of MRTK samples was calculated using the OCLR algorithm. The correlations of tumor stem cell index and the prognostic factors were analyzed by the Spearman correlation analysis. Additionally, we searched the target drugs of these prognostic factors from The Comparative Toxicogenomics Database (CTD). The interactions of target drugs and prognostic genes were visualized by Cytoscape software.

### Clinical Samples

This study has been approved by the Ethics Committee of Kunming Children’s Hospital, and all patients and their parents signed informed consent before joining the study. A total of six cases of fresh tumor and paratumor tissues were collected from children with MRTK admitted to Kunming Children’s Hospital from July 2014 to June 2020. There were 2 male and 4 female cases aging from 4 months to 4 years and were not treated with radiotherapy before surgery. The tumor tissues were excised as soon as possible after isolation in 0.5 cm^3^ volume, and relatively normal kidney tissues were taken from the distal cut edge of pathologically confirmed paratumor-infiltrated tissues. The samples were snap-frozen with liquid nitrogen and transferred to −80 °C for storage prior to use.

### Real-Time Reverse Transcription Polymerase Chain Reaction

Total RNA was extracted from MRTK tumor and paratumoral tissues using a TRIzol (T9424, Sigma, Germany) reagent following the manufacturer’s instructions. The RNA concentration and purity were determined using a NanoDrop 2000 spectrophotometer (Thermo Fisher Scientific). Then, mRNA was reversely transcribed into cDNA using a PrimeScript RT reagent kit with gDNA Eraser (RR047A, Takara, Japan). qRT-PCR was performed using a GoTaq qPCR and RT-qPCR kit (A6001, Promega, United States) using an ABI ViiATM7 real-time PCR system (Applied Biosystems, United States). The PCR program was started with an initial step of 95°C for 3 min, followed by 40 cycles of 15 s denaturation at 95°C and 30 s extension at 60°C. The mRNA expression levels were calculated by the 2^–ΔΔt^ method [△△ Ct = (Ct target gene-Ct internal reference gene) experimental group-(Ct target gene-Ct internal reference gene) control group] and normalized to GAPDH. The experiments were performed in triplicate for at least three samples of each group. The primers were synthesized by Sangon Biotech (Shanghai) and are listed in Table 1.

**TABLE 1:** Primer sequences of P4HA1, MLLT11, AURKA, GOT1, and GAPDH.

	Primer sequence (5′-3′)
P4HA1	Forward:AGTACATGACCCTGAGACTGGAAAA
	Reverse:ATCTGGCTCATCTTTCCGTGC
MLLT11	Forward:CCATCTTTGGAACACGCCAG
	Reverse:AACGCTGCTGTCTTTGACCT
AURKA	Forward:TGGAAGACTTGGGTCCTTGG
	Reverse:AAATATCCCCGCACTCTGGC
GOT1	Forward:GCCAAGTGGTCGAATCAACG
	Reverse:CTGGACGGGTGGTGTTTCTT
GAPDH	Forward:AGGTCGGTGTGAACGGATTTG
	Reverse:GGGGTCGTTGATGGCAACA

### Western Blot

The protein was extracted from tumor and paratumor tissues using RIPA buffer (Beyotime, Shanghai, China). BCA protein quantification kit (Shanshan Jinqiao Company, Beijing, China) was used for the quantification of protein samples. About 30 μg protein of each sample was loaded on SDS-PAGE gels. After transferring to the polyvinylidene fluoride membrane (Millipore, United States), the blots were probed with the appropriate primary antibody (anti-P4HA1, 1:1000, ProteinTech; anti-MLLT11, 1:1000, ProteinTech; anti-AURKA, 1:1000, Wanleibio; anti-GOT1, 1:1000, Wanleibio; anti-β-actin, 1:1000, Zen-Bioscience). After incubation with the primary antibodies, the membrane was incubated with the corresponding secondary antibodies and monitored with an enhanced chemiluminescence (ECL, Affinity, United States) substrate kit (Amersham, Biosciences, United States). The protein bands were photographed using an Image Lab system (Bio-Rad Laboratories, Inc., United States). After developing, the image was saved, and the Image Lab (Bio-Rad Laboratories, Inc., United States) analyzed the gray value.

### Statistical Analysis

All bioinformatics analyses involved in this research were conducted using the R software (version 3.6.3). The relationship between risk score and clinicopathological features was demonstrated using the Chi-square test. Moreover, the independent prognostic factors for MRTK identified the clinicopathological features by univariate and multivariate Cox regression analyses. GraphPad8.0 statistical software was used for data analysis of mRNA and protein expression. The t-test was used to compare the difference between the tumor and paratumor groups. *p* < 0.05 was considered statistically significant unless specified.

## Results

### Screening of Differentially Expressed mTORC1 Pathway-Related Genes in Malignant Rhabdoid Tumor of the Kidney

**FIGURE 1:**
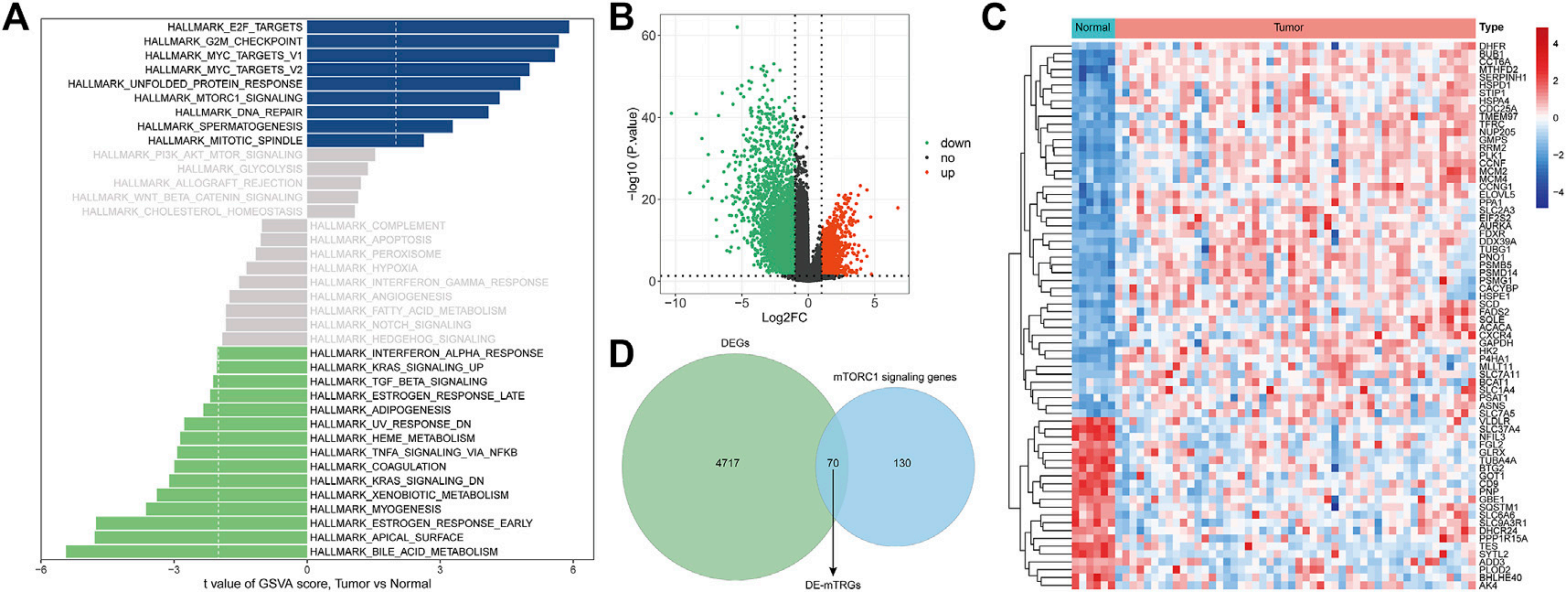
Identification of differentially expressed mTORC1 pathway-related genes (DE-mTRGs) in MRTK. **(A)** GSVA analysis; **(B)** volcano map. Green: downregulated (Down), red: upregulated (Up), black: unchanged (no); **(C)** Heatmap of DE-mTRGs in MRTK; **(D)** Venn diagram. Green: differentially expressed genes; blue: mTORC1 signaling genes;intersecting section: DE-mTRGs.

Through GSVA analysis, we screened 9 activated pathways in the MRTK group and 15 activated pathways in the normal group. Interestingly, we observed that the mTORC1 signaling pathway was activated in the MRTK samples (Figure 1A; Supplementary Table S1). Subsequently, we extracted the genes from the mTORC1 signaling pathway to screen the DEGs between the MRTK and normal samples. As a consequence, a total of 4787 DEGs were screened in the MRTK samples compared with normal samples, of which 1294 were upregulated and 3493 were downregulated (Figure 1B; Supplementary Table S2). After intersection with the 200 genes of the HALLMARK_MTORC1_SIGNALING gene set obtained from the MsigDB, we identified 70 DE-mTRGs in MRTK (Figure 1C). The expressions of 70 DE-mTRGs between the MRTK and normal samples were displayed in a heatmap. Among the 70 DE-mTRGs, 22 genes were downregulated and 48 genes were upregulated in patients with MRTK (Figure 1D).

### Development of a DE-mTRG-Related Signature in Malignant Rhabdoid Tumor of the Kidney

**FIGURE 2:**
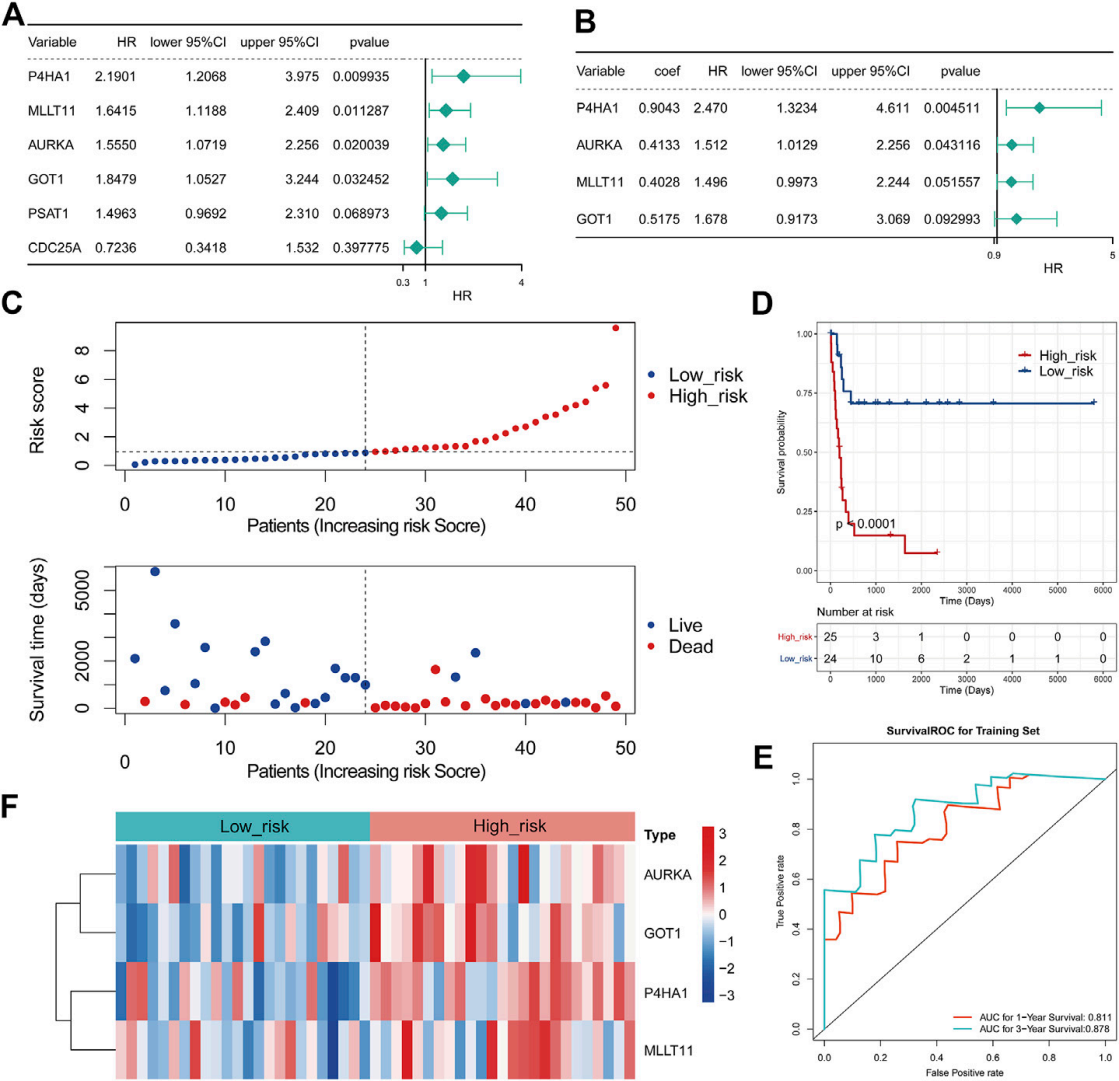
Development of an mTRG-related gene signature in MRTK. **(A)** Forest plot of hazard ratios (HRs) demonstrating the prognostic values of DE-mTRG. **(B)** Forest plot illustrating the multivariable Cox model results of each gene in the 4-mTRG risk signature. The dashed line was used to mark the location of HR = 1. **(C)** Relationship between the survival status/risk score rank and survival time (days)/risk score rank. **(D)** Kaplan–Meier (K-M) plot for overall survival (OS) based on the risk score of the four gene-based signature of patients with MRTK. **(E)** Receiver operating characteristic (ROC) curve for OS of the TARGET-MRTK set. The area under curve (AUC) was assessed at 1 and 3 years. **(F)** Heatmap demonstrating the distribution of the four DE-mTRG expressions in the TARGET-MRTK cohort.

To assess whether these 70 DE-mTRGs were related to survival, we first performed the K-M survival curves and univariate Cox regression analysis. As a result, four DE-mTRGs were demonstrated to be related to the OS of MRTK (Figure 2A, Supplementary Table S3, *p* < 0.05). A total of four DE-mTRGs including P4HA1, MLLT11, AURKA, and GOT1 were obtained from the multivariate Cox regression analysis, which were used to develop an mTRG-related gene signature (Figure 2B). Moreover, the survival analyses of each gene uncovered that patients with high expression of these genes were all related to a poor prognosis (all with *p* < 0.05, Supplementary Figure S1). The risk score of each MRTK patient was obtained as the formula mentioned in Materials and Methods. The MRTK patients were stratified into a high-risk group (*n* = 25) and a low-risk group (*n* = 24) by the boundary of the median risk score. The risk curves and patient survival scatter plots showed that the number of patient deaths increased with the risk score (Figure 2C). The K-M curves demonstrated that the risk score significantly differentiated clinical outcomes in MRTK patients (*p* < 0.0001), with a high-risk score implying a poor prognosis (Figure 2D). The area under curve (AUC) was 0.811 and 0.878 at 1 and 3 years, respectively, indicating the validity of the 4-gene-based prognostic signature (Figure 2E). Furthermore, the heatmap showed that all 4 model genes possessed relatively high expression levels in the high-risk group (Figure 2F).

### Independent Prognostic Analysis in Malignant Rhabdoid Tumor of the Kidney

**FIGURE 3:**
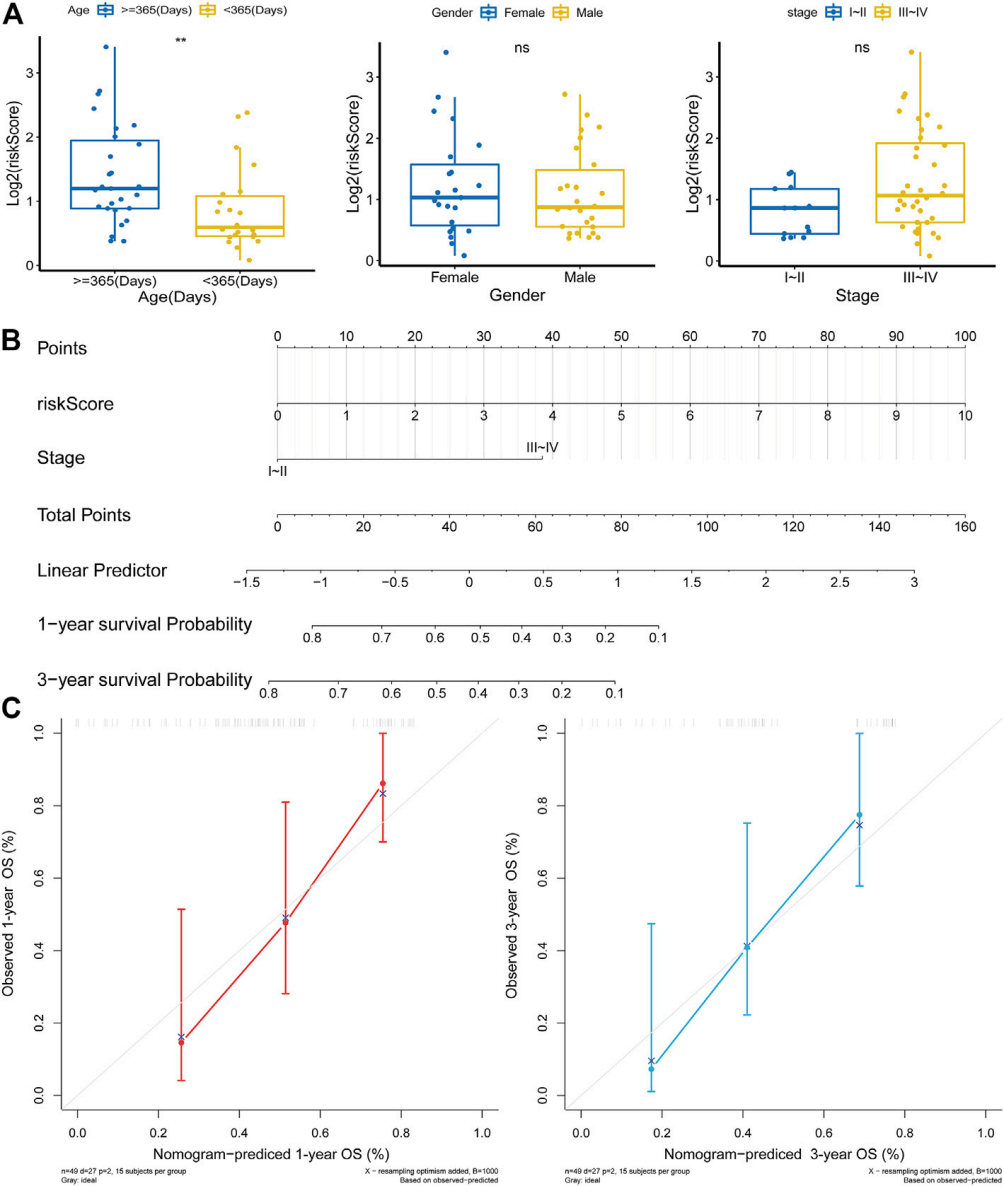
Independent prognostic analysis in MRTK. **(A)** Correlation analysis between risk score and clinicopathological characteristics. **(B)** Nomogram that integrated the risk score and stage predicted the probability of the 1- and 3-year OS. To calculate the survival probability, identification of the patient values on each axis and then for a vertical line upward to the point axis was drawn for each value. The points for all variables were added, and this sum on the total point line was located. **(C)** Calibration plot of the nomogram for predicting probabilities of 1-year and 3-year OS of patients.

Next, a Chi-square test was implemented to assess the distribution of clinical characteristics of MRTK patients in the high- and low-risk groups. The results showed that the age distribution of patients in the high- and low-risk groups was significantly different (*p* = 0.032), with 72.0% of patients in the high-risk group being ≥365 days old and 62.5% of patients in the low-risk group being less than 365 days old (Table 2). Furthermore, the Wilcoxon rank sum test indicated that the level of the risk score was significantly higher in MTRK patient’s ≥365 days than in MTRK patients’ <365 days (*p* < 0.01); however, the risk score was not statistically significant in the gender and stage subgroups (Figure 3A). Subsequently, we performed an independent prognostic analysis by Cox regression to assess whether the risk score could affect the clinical outcome of OS in MRTK patients independent of the clinicopathological characteristics (age, sex, and stage). The univariate Cox regression analysis showed that the risk score and stage were significantly associated with OS in patients with MRTK (*p* < 0.05; Table 3). Finally, multivariate Cox regression analysis pointed out that the risk score and stage were independent prognostic factors for MRTK patients (Table 4). Thereafter, we constructed a nomogram based on two independent prognostic factors that predicted patients’ 1-year and 3-year OS (Figure 3B), which had a C-index of 0.767. The calibration curve indicated that the predictive value of the nomogram model for OS in MRTK patients was similar to the actual observed value (Figure 3C), implying that the combined model of the two independent prognostic factors may be more clinically applicable.

**TABLE 2:** High and low risk groups association with clinical characteristics.

	Expression			
	Total (*N* = 49)	High_risk (*N* = 25)	Low_risk (*N* = 24)	p-value
Gender				
Female	23 (46.9%)	13 (52.0%)	10 (41.7%)	0.661
Male	26 (53.1%)	12 (48.0%)	14 (58.3%)	
Age(Days)				
≥365(Days)	27 (55.1%)	18 (72.0%)	9 (37.5%)	0.032
<365(Days)	22 (44.9%)	7 (28.0%)	15 (62.5%)	
Pathologic stage				
I-II	13(26.5%)	4 (16.0%)	9 (37.5%)	0.167
III-IV	36 (73.5%)	21 (84.0%)	15(62.5%)	

**TABLE 3:** Univariate Cox regression analysis of high-risk and low-risk groups.

ID	HR	HR.95L	HR.95H	p-value
riskScore	1.39	1.19	1.61	0.00
Stage(Reference:StageI∼II)	3.90	1.17	12.98	0.03
Gender(Reference:Female)	0.63	0.29	1.36	0.24
Age_at_Diagnosis_in_Days	1.00	1.00	1.00	0.25

**TABLE 4:** Multivariate Cox regression analysis of high-risk and low-risk groups.

ID	HR	HR.95L	HR.95H	p-value
riskScore	1.34	1.15	1.56	0.00
Stage(Reference:StageI∼II)	3.05	0.89	10.42	0.08

### Gene Set Variation Analysis of the Prognostic Biomarkers

**FIGURE 4:**
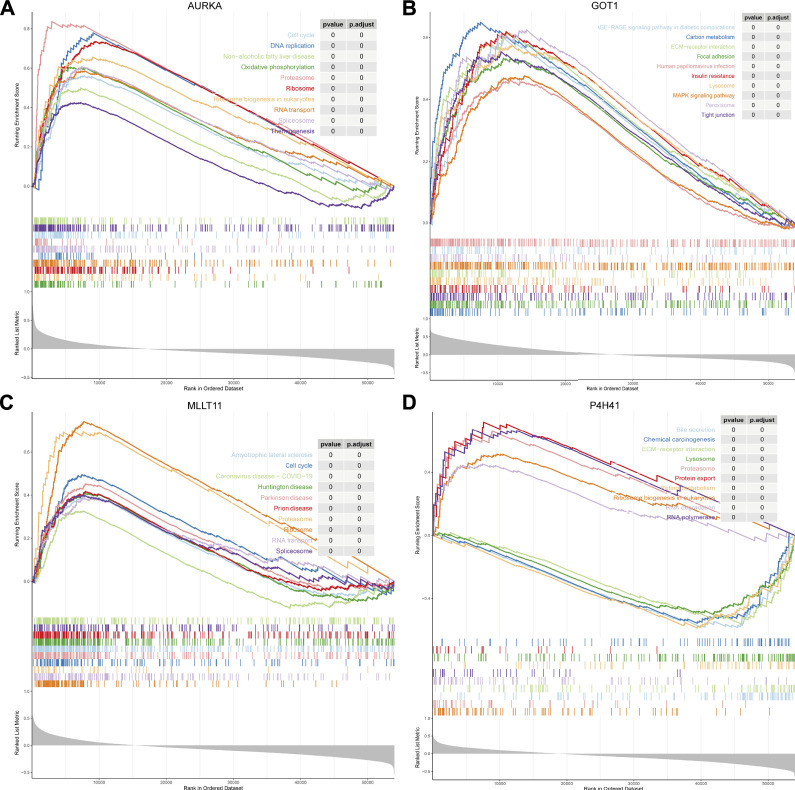
GSEA of the prognostic biomarkers. GSEA explores related signal pathways of **(A)** AURKA; **(B)** GOT1; **(C)** MLLT11; and **(D)** P4H41.

We conducted GSEA to further explore the involved signaling pathways of the four prognostic biomarkers. The results indicated that AURKA was mainly involved in cell cycle, DNA replication, non-alcoholic fatty liver disease, oxidative phosphorylation, proteasome, ribosome, and ribosome biogenesis in eukaryotes, RNA transport, spliceosome, and thermogenesis (Figure 4A; Supplementary Table S4). Similarly, GOT1 was significantly associated with various signaling pathways such as ECM–receptor interaction, carbon metabolism, human papillomavirus infection, AGE–RAGE signaling pathway in diabetic complications, focal adhesion, insulin resistance, lysosome, MAPK signaling pathway, peroxisome, and tight junction (Figure 4B; Supplementary Table S5). Interestingly, the expression of MLLT11 was correlated with several diseases, including amyotrophic lateral sclerosis, coronavirus disease-COVID-19, Huntington’s disease, Parkinson’s disease, and prion disease (Figure 4C; Supplementary Table S6). In addition, P4H41 was also mainly involved in bile secretion, chemical carcinogenesis, ECM–receptor interaction, lysosome, proteasome, RNA degradation, and RNA polymerase (Figure 4D; Supplementary Table S7).

### Functional Enrichment Analysis

**FIGURE 5:**
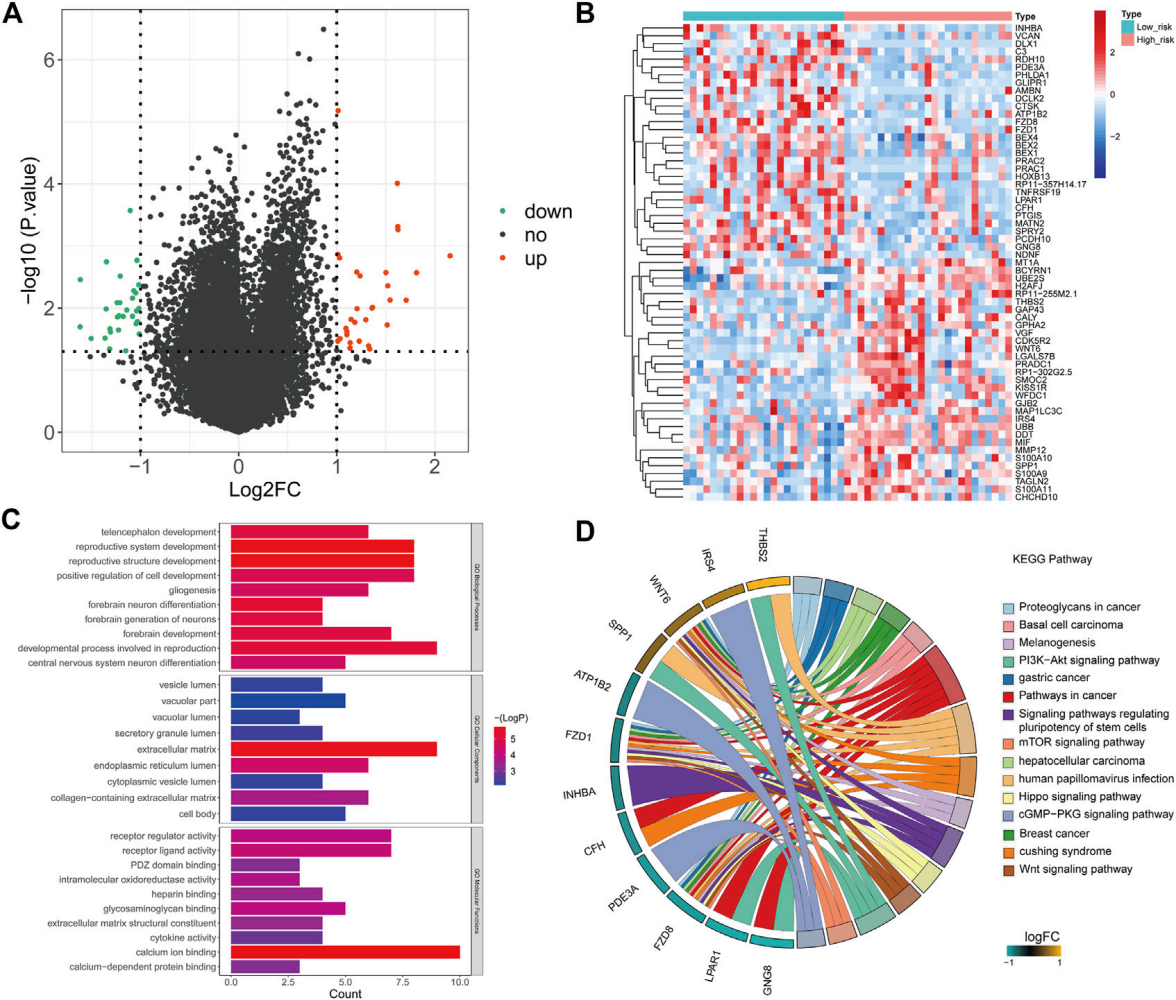
Functional enrichment analysis. **(A)** Volcano plot showing the DEGs of the risk score (high vs. low). Green: downregulated (Down), red: upregulated (Up), black: unchanged (no). **(B)** Heatmap based on 61 DEGs that were differentially expressed between high- and low-risk score samples. Each column represents a sample, and each row represents the mRNA level. Red indicates that the gene is upregulated, and green indicates downregulated genes. **(C)** Top 10 Gene Ontology terms enriched by the DEGs. **(D)** KEGG enrichment analysis of DEGs. The left side of the circle is the gene, and the right side is the pathway. Different colors represent different pathways, and the color of each pathway is annotated in right of the circle. If the gene belongs to a pathway, there will be a line between the gene and the pathway.

To elucidate the molecular mechanism that was involved in the risk score, we conducted GO and KEGG functional enrichment analyses on DEGs of the two risk groups (Figures 5A,B). The results showed that the DEGs mainly focused on several important biological processes including central nervous system neuron differentiation and developmental process involved in reproduction, forebrain development, forebrain neuron differentiation, positive regulation of cell development, reproductive structure development, and reproductive system development (Figure 5C). KEGG analysis indicated that these DEGs were mainly associated with basal cell carcinoma, pathways in cancer, proteoglycans in cancer, melanogenesis, PI3K–AKT signaling pathway, gastric cancer, signaling pathways, mTOR signaling pathway, regulating pluripotency of stem cells, hepatocellular carcinoma, human papillomavirus infection, Hippo signaling pathway, cGMP-PKG signaling pathway, breast cancer, Cushing syndrome, and Wnt signaling pathway (Figure 5D).

### Target Drug Prediction Based on the Tumor Stem Cell Index

**FIGURE 6:**
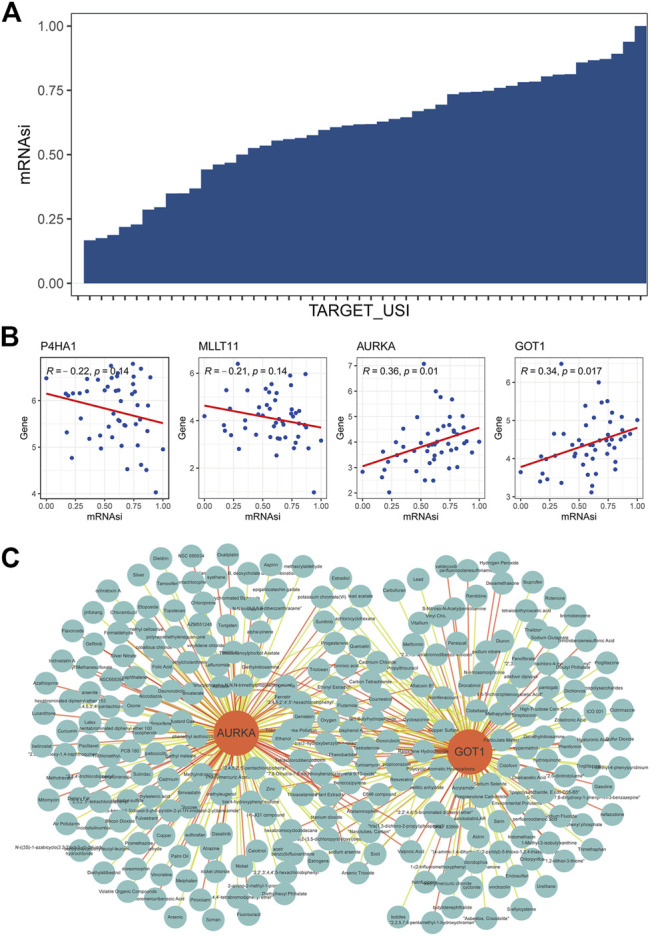
Target drug prediction based on the tumor stem cell index. **(A)** Tumor stem cell index (mRNAsi) of MRTK samples based on the OCLR algorithm. TARGET_USI: unique sample identity document (USI) for 50 MRTK samples from the TARGET database; **(B)** correlation analysis between mRNAsi and P4HA1, MLLT11, AURKA, and GOT1; **(C)** Interaction between target drugs and prognostic factors that are positively correlated with mRNAsi effect.

It has been reported that mTOR exerts important functions in cancer stem cells through the specific functions related to stemness (Matsubara et al., 2013). We calculated the tumor stem cell index (mRNAsi) of MRTK samples based on the OCLR algorithm (Zhang et al., 2020) (Figure 6A). The correlation analysis showed that the expressions of AURKA and GOT1 were positively correlated with the mRNAsi in MRTK (Figure 6B). Then, we searched the corresponding target drugs of AURKA and GOT1 from the CTD. A network containing 262 nodes and 349 edges showed the interactions between the target drugs and prognostic factors that were positively correlated with mRNAsi (Figure 6C). These target drugs predicted by AURKA and GOT1 may play an essential role in the therapy of MRTK.

### Expression Validation of Prognostic Biomarkers in Clinical Samples

**FIGURE 7:**
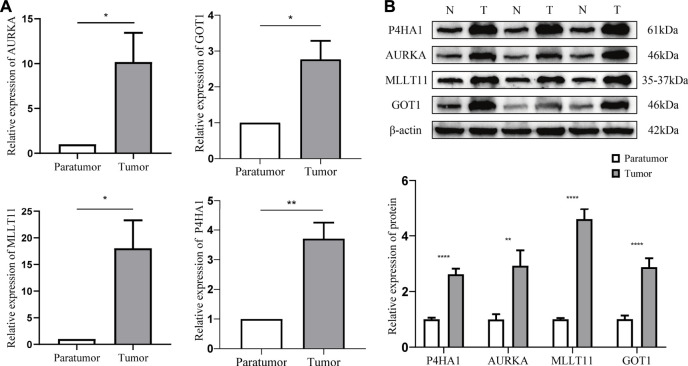
Expression validation of prognostic biomarkers in clinical samples. **(A)** Expressions of P4HA1, MLLT11, AURKA, and GOT1 in tumor and paratumor samples by RT-qPCR. **(B)** Expressions and quantification result of P4HA1, MLLT11, AURKA, and GOT1 in tumor and paratumor samples by Western blot. *, *p* < 0.05 vs. control; **, *p* < 0.01 vs. control; ***, *p* < 0.001; ****, *p* < 0.0001.

RT-qPCR results showed that the abundance of P4HA1, MLLT11, AURKA, and GOT1 were significantly elevated in tumor tissues (Figure 7A). Consistent with the RT-qPCR results, Western blot also showed that the protein levels of P4HA1, MLLT11, AURKA, and GOT1 were markedly higher in the tumor tissues than those in the paratumor tissues (Figure 7B). These results were in accordance with the RNA-sequencing results, indicating the reliability of our analysis.

## Discussion

In this study, we first determined that the mTORC1 signaling pathway was activated in the MRTK samples through GSVA analysis. Tuberous sclerosis, a disease in which mTORC1 is over-activated by TSC1 or TSC2 deletion, which leads to the formation of a wide range of benign tumors, is also direct evidence that mTORC1 plays a key role in tumorigenesis. The negative feedback of insulin receptor substrate (IRS)-1 mediated by mTORC1 may be the reason for the limited progression of these tumors. It can effectively downregulate PI3K signaling downstream of most receptor tyrosine kinases (RTKs) (Harrington et al., 2004; Shah et al., 2004; Um et al., 2004). In addition, mTORC1 can directly phosphorylate a linker called Grb10 that directly binds to RTK (Hsu et al., 2011; Yu et al., 2011). The patients with tuberous sclerosis also proved that mTORC1 signaling as a single molecular mutation is a powerful driving force for cell proliferation. Under the premise of genetic and molecular changes, mTORC1 signaling aggravates tumor progression through a variety of molecular mechanisms.

Autophagy is an intracellular process that leads to the orderly degradation and recycling of cellular components. In addition to regulating cell growth and metabolism, mTORC1 also regulates this process of autophagy. mTORC1 can negatively regulate autophagy inhibition to initiate autophagy through the phosphorylation of ULK and VPS34 (Kim and Guan, 2015). The knockout of Beclin1 can block autophagy and promote tumor formation, so autophagy is considered to be an effective tumor suppressor (Qu et al., 2003; Yue et al., 2003; White, 2015). However, there is a lot of evidence that tumor cells can also use autophagy to enhance cell activity under metabolic stress (Degenhardt et al., 2006). In this case, the inhibition of mTORC1 will greatly enhance autophagy, which may cause cells to increase the synthesis of nutrient molecules and enhance tumor cell survival.

Based on mTORC1-related genes, we constructed a prognostic model by 4 gene signatures, including P4HA1, MLLT11, AURKA, and GOT1. Collagen prolyl 4-hydroxylase alpha polypeptide I (P4HA1) is essential for the collagen prolyl hydroxylation of normal cells. Previous studies on P4HA1 mainly focused on the congenital connective tissue disorder caused by gene mutation, and the correlation between P4HA1 and tumors has rarely been reported. In recent years, the relationship between P4HA1 and tumors has attracted people’s attention. Gilkes et al. (2013) found that the hypoxia-inducible factor (HIF1) was widely involved in ECM remodeling by regulating P4HA1 and P4HA2, thereby regulating the occurrence and development of breast cancer. *In vivo* and *in vitro* studies by Chakravarthi et al. (2014) showed that the over-expression of P4HA1 improved the proliferation and invasion ability of prostate cancer cells through the miR-124/P4HA1/MMP1 axis. A new study indicated that miR-122 inhibited the epithelial–mesenchymal transformation process by targeting P4HA1, thereby regulating the invasion and abdominal metastasis of ovarian cancer (Zhou et al., 2017; Duan et al., 2018). Aurora kinase A (AURKA) is a member of the serine/threonine kinase family necessary for the regulation of mitosis and the process of cell division. Aurora kinase A (AURKA) can regulate the process of mitosis, centrosome maturation and separation, and spindle mitosis. (Marumoto et al., 2005). Due to the extremely high expression of AURKA in cancer, whether AURKA can be used as a potential therapeutic target has aroused great interest in the academic community (Marumoto et al., 2005). Aurora kinase inhibitors such as MLN8237 and PHA-739358 have been developed and applied (Kollareddy et al., 2012), but only moderate effects have been found in preclinical and clinical studies (Kollareddy et al., 2012; Goldberg et al., 2014). In addition, new evidence shows that AURKA also promotes the occurrence and development of cancer through other mechanisms unrelated to its kinase activity (Otto et al., 2009). Glutamine metabolism is also very important for the proliferation of cancer cells. Recent studies have shown that hyperproliferative cells can use glutamine derived from glutamine to produce non-essential amino acids (NEAA) through glutamate-oxaloacetate transaminase (*GOT1*), but resting cells can pass through glutamate dehydrogenase 1 (*GLUD1*) and the subsequent decarboxylation reaction in the TCA cycle of metabolizing glutamate (Hensley et al., 2013). Research by Fenja M Feld et al. proved that *GOT1* could be used as a prognostic biomarker for pancreatic ductal cancer (Yang, 2016). Yang Yong et al. have shown that inhibiting *GOT1* can enhance the efficacy of adriamycin against triple-negative breast cancer (Yang, 2016). The MLLT11 gene, located on chromosome 1 band q21, was initially identified as a mixed-lineage leukemia (MLL) fusion partner from acute myeloid leukemia (AML) patients whose leukemic cells carried a t(1; 11) (q21; q23) chromosomal abnormality (Tse et al., 1995). The expression of MLLT11 is strictly regulated in normal lineage-directed hematopoietic progenitor cells (HPCs) (Tse et al., 1995). However, the expression of MLLT11 is increased widely in acute myeloid and lymphoid leukemia (Tse et al., 1995). Research by Yin Xiong et al. proved that AML children with low MLLT11 expression have poorer overall survival (Xiong et al., 2011). However, Elisabeth S Gruber proved that the high expression of AF1q (MLLT11 corresponding gene name) could independently be used as a prognostic indicator for patients with esophageal cancer (Gruber et al., 2019).

Subsequently, we performed a functional enrichment analysis on the DEGs of the high- and low-risk groups. Interestingly, the mTOR signaling pathway was significantly enriched. Previous studies have shown the importance of the mTOR pathway in the pathogenesis of cancer. mTOR complex 1 (mTORC1) can increase mRNA translation, protein synthesis, and cell proliferation (Tasian et al., 2014). The activation of the second mTOR complex (mTORC2) involved in the regulation of the cytoskeleton may be a feedback effect of the AKT loop (Tasian et al., 2014). Balsara et al. found that 74% of specimens from patients with non-small cell lung cancer (NSCLC) stained positive for mTOR through the use of tissue microarrays (TMAs) (Balsara et al., 2004). Rictor is a subunit of mTORC2, which promotes the assembly and activity of mTORC2, and endows glioma cells with proliferation and invasion potential (Fan and Weiss, 2012).

Cancer stem cells (CSCs) are a subgroup of tumor tissues that are highly immune to traditional cancer treatments. At present, slow-circulating CSC is the main obstacle to the eradication of most tumors (Begicevic and Falasca, 2017). Traditionally, the mTOR pathway is over-activated in CSC. Furthermore, it has been found that targeted inhibition of the mTORC1 signaling pathway is 30 times more powerful than non-CSC in inhibiting the proliferation and survival of CSC in solid tumor cell populations. Transforming growth factor-*β* (TGF-*β*) can promote epithelial–mesenchymal transition (EMT), thereby increasing the production and activation of cancer stem cells. mTOR is a key node in the TGF-*β* signaling pathway to enhance cancer stemness and drug resistance (Katsuno et al., 2019). Some mTOR inhibitors have shown inhibitory function on CSC (Francipane and Lagasse, 2016), for example, rapamycin, everolimus, and PF-04691502 can inhibit the activation of breast cancer stem cells induced by tamoxifen (Karthik et al., 2015). Also, inhibiting mTOR can restore the resistance of breast cancer cells to tamoxifen (DeGraffenried et al., 2004). In addition, Torin1 (ATP competitive mTOR inhibitor) and VS-5584 (PI3K/mTOR inhibitor) can significantly reduce tumor CSC levels in a variety of human cancer transplant models (Francipane and Lagasse, 2013; Kolev et al., 2015). Increasing studies have demonstrated the role of the mTOR signaling pathway in maintaining CSC. Chang et al. (https://www.ncbi.nlm.nih.gov/pubmed/24157869/) found that the radiation resistance of prostate cancer was related to the enhanced CSC phenotype through the activation of the PI3K/AKT/mTOR signaling pathway (Chang et al., 2013). Inhibition of mTORC2 leads to a reduction in the expression of liver CSC markers (epithelial cell adhesion molecule, EpCAM) in hepatocellular carcinoma stem cells (Sunayama et al., 2010; Nishitani et al., 2013). Bleau et al. found that the cross-inhibitory regulation between MEK/ERK and PI3K/mTOR pathways maintained the self-renewal and tumorigenic ability of glioblastoma cancer stem-like cells (Bleau et al., 2009). Corominas-Faja et al. found that AKT regulated the activity of the ATP-binding cassette transporter (ABCG2) in glioma tumor stem-like cells (Corominas-Faja et al., 2013).

Our study only analyzed the data of TCGA database, and the results have several limitations. In our subsequent studies, we will collect clinical specimens combining cells and animal experiments to further verify the results in our study, which provides a reliable theoretical basis for the treatment of MRTK. Also, the reliability of the results was demonstrated by RT-PCR and WB validation of fresh tumor tissue and paratumor samples from six children.

The US Food and Drug Administration (FDA) approved an mTOR inhibitor for the treatment of renal cell carcinoma, named temsirolimus (Bergmann et al., 2014). So far, humans have developed three generations of compounds targeting the PI3K/mTOR signaling pathway. As the first generation of PI3K inhibitors, pan-inhibitors can be used to bind to all PI3K targets (Martelli et al., 2012). The second-generation inhibitors with isoform-specific selective activity have higher specificity (Martelli et al., 2012). The dual PI3K/mTOR inhibitor is the third-generation inhibitor. It can inhibit not only all PI3K I subtypes but also mTORC1 and mTORC2 (Zhu et al., 2012).

## Conclusion

In conclusion, we found that the mTORC1 signaling pathway was activated in MRTK samples and successfully constructed a risk model using differentially expressed mTORC1 pathway genes. A total of four biomarker genes, P4HA1, MLLT11, AURKA, and GOT1, were selected. The potential molecular mechanisms of the four biomarker genes involved in MRTK and their correlation with the tumor stem cell index were explored. Target drugs of AURKA and GOT1 were predicted, which need further experimental screening. Our study provides a reference for further understanding the possible pathogenesis in the prognosis of MRTK.

## Data Availability

The datasets presented in this study can be found in online repositories. The names of the repository/repositories and accession number(s) can be found in the article/Supplementary Material.
