# Supplementary figures and images for: Identification of Prognostic Biomarkers in Patients With Malignant Rhabdoid Tumor of the Kidney Based on mTORC1 Signaling Pathway-Related Genes
Source: Front Mol Biosci. 2022 Apr 26;9:843234. doi: 10.3389/fmolb.2022.843234 (PMC9087638; doi:10.3389/fmolb.2022.843234)

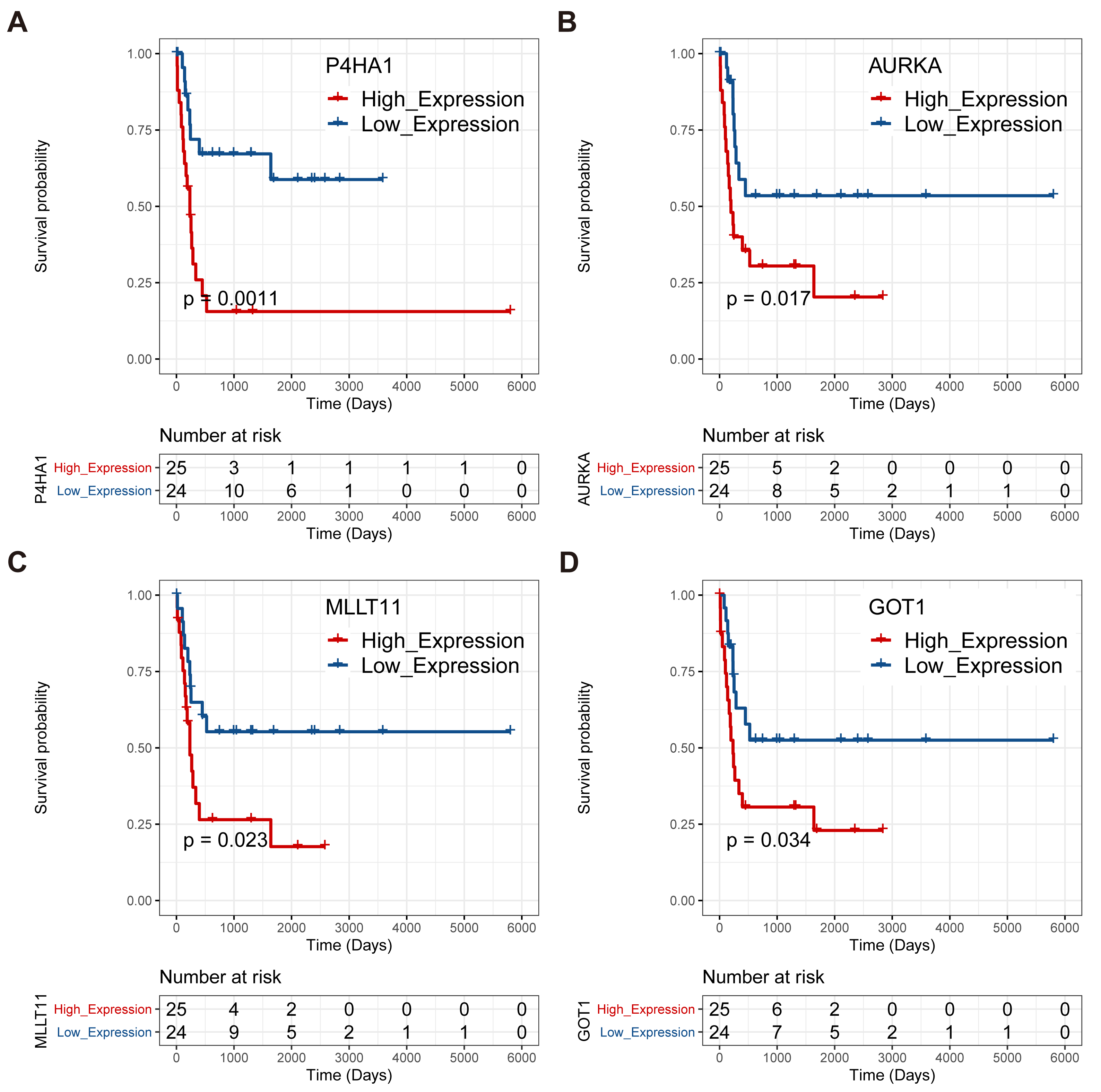

Supplement: Supplementary file 6 [file Image1.TIF]
